# Identification of Cortical and Subcortical Correlates of Cognitive Performance in Multiple Sclerosis Using Voxel-Based Morphometry

**DOI:** 10.3389/fneur.2018.00920

**Published:** 2018-10-29

**Authors:** Jordi A. Matías-Guiu, Ana Cortés-Martínez, Paloma Montero, Vanesa Pytel, Teresa Moreno-Ramos, Manuela Jorquera, Miguel Yus, Juan Arrazola, Jorge Matías-Guiu

**Affiliations:** ^1^Department of Neurology, San Carlos Institute for Health Research, Hospital Clinico San Carlos, Universidad Complutense de Madrid, Madrid, Spain; ^2^Department of Radiology, San Carlos Institute for Health Research, Hospital Clinico San Carlos, Universidad Complutense de Madrid, Madrid, Spain

**Keywords:** multiple sclerosis, cognitive impairment, voxel-based morphometry, magnetic resonance imaging, neuropsychological assessment, memory, executive functioning

## Abstract

**Objective:** Cognitive impairment is an important feature in multiple sclerosis (MS) and has been associated to several Magnetic Resonance Imaging (MRI) markers, but especially brain atrophy. However, the relationship between specific neuropsychological tests examining several cognitive functions and brain volumes has been little explored. Furthermore, because MS frequently damage subcortical regions, it may be an interesting model to examine the role of subcortical areas in cognitive functioning. Our aim was to identify correlations between specific brain regions and performance in neuropsychological tests evaluating different cognitive functions in a large series of patients with MS.

**Methods:** A total of 375 patients were evaluated with a comprehensive neuropsychological battery and with MRI. Voxel-based morphometry was conducted to analyse the correlation between cognitive performance and gray matter damage, using Statistical Parametric Mapping with the toolboxes VBM8 and Lesion Segmentation Tool.

**Results:** The following correlations were found: Corsi block-tapping test with right insula; Trail Making Test with caudate nucleus, thalamus, and several cortical regions including the posterior cingulate and inferior frontal gyrus; Symbol Digit Modalities Test with caudate nucleus, thalamus, posterior cingulate, several frontal regions, insula, and cerebellum; Stroop Color and Word Test with caudate nucleus and putamen; Free and Cued Selective Reminding Test and Rey-Osterrieth Complex Figure with thalamus, precuneus, and parahippocampal gyrus; Boston Naming Test with thalamus, caudate nucleus, and hippocampus; semantic verbal fluency with thalamus and phonological verbal fluency with caudate nucleus; and Tower of London test with frontal lobe, caudate nucleus, and posterior cingulate.

**Conclusion:** Our study provides valuable data on the cortical and subcortical basis of cognitive function in MS. Neuropsychological tests mainly assessing attention and executive function showed a stronger association with caudate volume, while tests primarily evaluating memory were more strongly correlated with the thalamus. Other relevant regions were the posterior cingulate/precuneus, which were associated with attentional tasks, and several frontal regions, which were found to be correlated with planning and higher order executive functioning. Furthermore, our study supports the brain vertical organization of cognitive functioning, with the participation of the cortex, thalamus, basal ganglia, and cerebellum.

## Introduction

Multiple sclerosis (MS) is an acquired, demyelinating, and neurodegenerative disease of the central nervous system. Cognitive impairment is frequent and involves multiple functions, particularly attention and executive function, memory, and information processing speed (1). However, other cognitive domains may also be impaired, which explains the marked heterogeneity of these patients' cognitive profiles (2).

Cognitive impairment has been associated with several MRI markers, including brain atrophy, lesion burden, lesions to white matter tracts, or normal-appearing white matter damage. Cognitive impairment has traditionally been considered to be more closely related to brain atrophy than to white matter lesion burden (3). Other studies have associated cognitive dysfunction with other MRI findings, such as the number of cortical lesions, magnetization transfer ratio in gray matter, and white matter damage in diffusion tensor imaging (4–7).

Most of these studies have used global brain measures (8). In this regard, several studies have attempted to define neuroimaging markers in order to predict the presence of cognitive impairment (9, 10). For instance, Calabrese et al. (11) found that cortical lesion and normalized neocortical gray matter volumes were independent predictors of cognitive impairment in relapsing remitting MS. However, last investigations suggest that regional volumes could be better predictors of cognitive impairment than whole-brain MRI measurements. Accordingly, some studies have estimated regression models to predict the presence of cognitive impairment, generally including thalamic volume and other factors such as age, sex, EDSS, or years of education (12–14).

Conversely, few studies have investigated the relationship between particular cognitive deficits (e.g., memory or visuospatial impairment) and regional brain volume or cortical thickness. These studies usually include relatively small samples or focus on specific tests or brain regions, sometimes with controversial results. For instance, Symbol Digit Modalities Test (SDMT), one of the most used neuropsychological tools in cognitive assessment in MS, showed no significant correlation with regional brain volumes in a case series of 15 patients (15), but correlated with prefrontal cortex, precentral and postcentral gyri, and right temporal cortex in a group of 18 patients with MS (16), and correlated with thalamus, cerebellum, putamen, and occipital cortex in a recent study including 125 patients (17).

Another issue of previous studies is that they mainly aimed to find neural correlates of global cognitive impairment, regarding this as a dichotomous feature, rather than evaluating specific cognitive domains or neuropsychological tests (8). The cognitive functions that have been most frequently studied are memory and information processing speed, while others such as executive functioning, language or visuospatial functioning have received much less attention (9, 17–19). Furthermore, very few studies have analyzed the neural correlates of a series of neuropsychological tests assessing different cognitive functions in one same sample, which would be more useful for understanding the neural basis of each cognitive domain rather than specific studies of one function or test in different samples.

Given the important role of brain atrophy in cognitive dysfunction associated with MS, we sought to identify correlations between atrophy in certain brain regions and the results from several neuropsychological tests using a large series of patients with MS. We used voxel-based morphometry (VBM) to determine the role of particular gray matter brain regions in the pathophysiology of cognitive impairment in MS. VBM provides highly consistent results and has been validated for studying the relationship between cognitive functions and brain regions (20), allowing an unbiased approach to brain-behavior correlations without an a priori hypothesis. Furthermore, MS may provide an interesting model for the study of the complex relationship between cognitive function and subcortical regions, due to the frequent involvement of subcortical gray and white matter in MS (21). Much emphasis has been placed in recent years on the role of subcortical structures in cognitive function, leading to a shift from a cortico-centric viewpoint (in which the cerebral cortex was considered the primary site of cognition) to a model also including the basal ganglia and cerebellum (22). Some studies of MS have found a correlation between atrophy of these structures and cognitive impairment. In this regard, the thalamus has been associated with cognitive impairment (23, 24), and thalamic atrophy may even be a sensitive marker of cognitive dysfunction (12).

## Methods

### Participants

All patients included in the study met the revised McDonald criteria for the diagnosis of MS (25). We excluded patients with potential causes of cognitive impairment other than MS (a history of alcohol or drug abuse, systemic or developmental disorders, major depressive disorder, etc.), or any condition that may bias cognitive assessment results (visual or motor impairment that may interfere with test completion, MS relapses in the previous 6 months, etc.). The study was approved by our hospital's ethics committee; written informed consent was obtained from all participants.

Our study included 375 patients, with a mean age of 47.15 ± 10.27 years, and 262 of them (69.9%) were women. Main demographic and clinical characteristics are shown in Table 1.

**Table 1:** Main demographic and clinical characteristics.

Age (years)	47.15 ± 10.27
Sex (% women)	262 (69.9%)
Years of education	15.15 ± 3.63
Clinical course	273 (72.8%) Relapsing-Remitting 73 (19.5%) Secondary Progressive 29 (7.7%) Primary Progressive
EDSS (median, interquartile range)	2.5 [1.5–4.0]
T2 lesion volume (mL)	17.3 ± 20.0
Brain parenchymal fraction	0.80 ± 0.02
Gray matter fraction	0.41 ± 0.03

### Neuropsychological assessment

All patients underwent a thorough cognitive assessment with the following tests: forward and backward digit span (26), Corsi block-tapping test (26), Trail Making Test (TMT) parts A and B (27), Symbol Digit Modalities Test (SDMT; written version) (28), Boston Naming Test (BNT) (29), Judgement of Line Orientation (JLO) (30), Rey-Osterrieth Complex Figure (ROCF; copy and recall at 3 and 30 min) (31, 32), Free and Cued Selective Reminding Test (FCSRT) (33, 34), verbal fluencies (animals and words beginning with “p,” “r,” and “s” in 1 min) (35), Stroop Color and Word Test (SCWT) (36), and Tower of London-Drexel version (ToL) (37).

These tests were selected for 3 main reasons: they are standardized, they assess key cognitive domains, and comprehensive normative data for each of them are available in our setting (38, 39). Following the normative data, we defined patients with cognitive impairment as those having alterations in at least two cognitive domains, using a percentile ≤ 5 in age- and education-adjusted scores as the cut-off point. This is explained in greater detail elsewhere (2).

Furthermore, patients were assessed for depression using the Beck Depression Inventory (40).

### MRI acquisition protocol

All patients underwent MRI scans. The following sequences were acquired:

a) T1-weighted 3D fast spoiled gradient-echo (FSPGR) inversion recovery. In 250 patients: repetition time [RT] 12 ms, echo time [ET] 2.3 ms, inversion time [IT] 400 ms; gap 0.0 mm, number of excitations [NEX] 1; acquisition matrix 256 × 192, field of view [FOV] 25 × 20 cm); slice thickness 3 mm (voxel size 1 × 1 × 3 mm); in 125 patients, RT 6.7 ms, ET 1.6 ms, slice thickness 1 mm (voxel size 1 × 1 × 1 mm).
b) T2-weighted fluid-attenuated inversion recovery (FLAIR) (RT 9,102 ms, ET 121 ms, IT 2,250 ms; slice thickness 3 mm; gap 0.4 mm; NEX 1; acquisition matrix 256 × 192; FOV 24 cm).

Images were acquired with a 1.5 T scanner (Signa HDxt, GE Healthcare, Milwaukee, United States). The time interval between MRI scans and cognitive assessment was <6 months.

### Image pre-processing and analysis

Images were pre-processed and analyzed using Statistical Parametric Mapping version 8 (SPM8) (The Wellcome Trust Center for Neuroimaging, Institute of Neurology, University College of London) with the VBM8 and Lesion Segmentation Tool (LST) toolboxes (41). White matter lesions were segmented using LST. This toolbox segments T2-hyperintense MS-associated white matter lesions using 3D-T1 and FLAIR sequences using a lesion growth algorithm (41). We used a threshold kappa-value of 0.3, as recommended; segmentations for all patients were also visually inspected. Lesion maps were used for filling T1-weighted images. Subsequently, 3D T1-weighted sequences from each patient were normalized using both linear and non-linear registration, then segmented into gray matter, white matter, and cerebrospinal fluid. Segmentation in VBM8 is based on the “new segment” procedure of SPM8, and uses an adaptive Maximum A Posterior technique and Partial Volume Estimation. Furthermore, it applies two denoising methods (an optimized block-wise non-local means denoising filter and Markov Random Field, and also integrates the Diffeomorphic Anatomical Registration Through Exponentiated Lie Algebra (DARTEL) normalization procedure (42). High dimensional DARTEL normalization was used to normalize images to the Montreal Neurological Institute space. Then, normalized gray matter images were modulated, by scaling with the Jacobian determinants, and finally smoothed with a Gaussian kernel of 8 mm full-width at half maximum (FWHM). The following estimation options were used (bias regularization 0.0001; FWHM of Gaussian smoothness of bias 60 mm cut-off; affine regularization: International Consortium for Brain Mapping space template European brains; sampling distance 3; Markof Random field weighting 0.15). We checked data quality by displaying images individually and using the VBM8 function “check sample homogeneity using covariance.” Lesion maps were used to calculate total lesion volume. Global structural volumes were estimated from the lesion filled T1-weighted images and normalized with the total intracranial volume. During the process of imaging analysis, five patients were excluded due to presence of artifacts or insufficient image quality.

### Statistical analysis

Statistical analysis was performed using IBM® SPSS Statistics version 20.0. Data are reported as means ± standard deviation (SD), median (interquartile range) and absolute frequencies (percentages).

Partial correlations between cognitive tests and global measures (lesion load and normalized gray matter volume) were calculated, controlling for age, sex, and years of education. Bonferroni correction was used to correct for multiple comparisons considering the number of cognitive tests. Correlations were interpreted as very weak (0–0.19), weak (0.20–0.39), moderate (0.40–0.59), strong (0.60–0.79), or very strong (0.80–1) (43).

**Figure 1:**
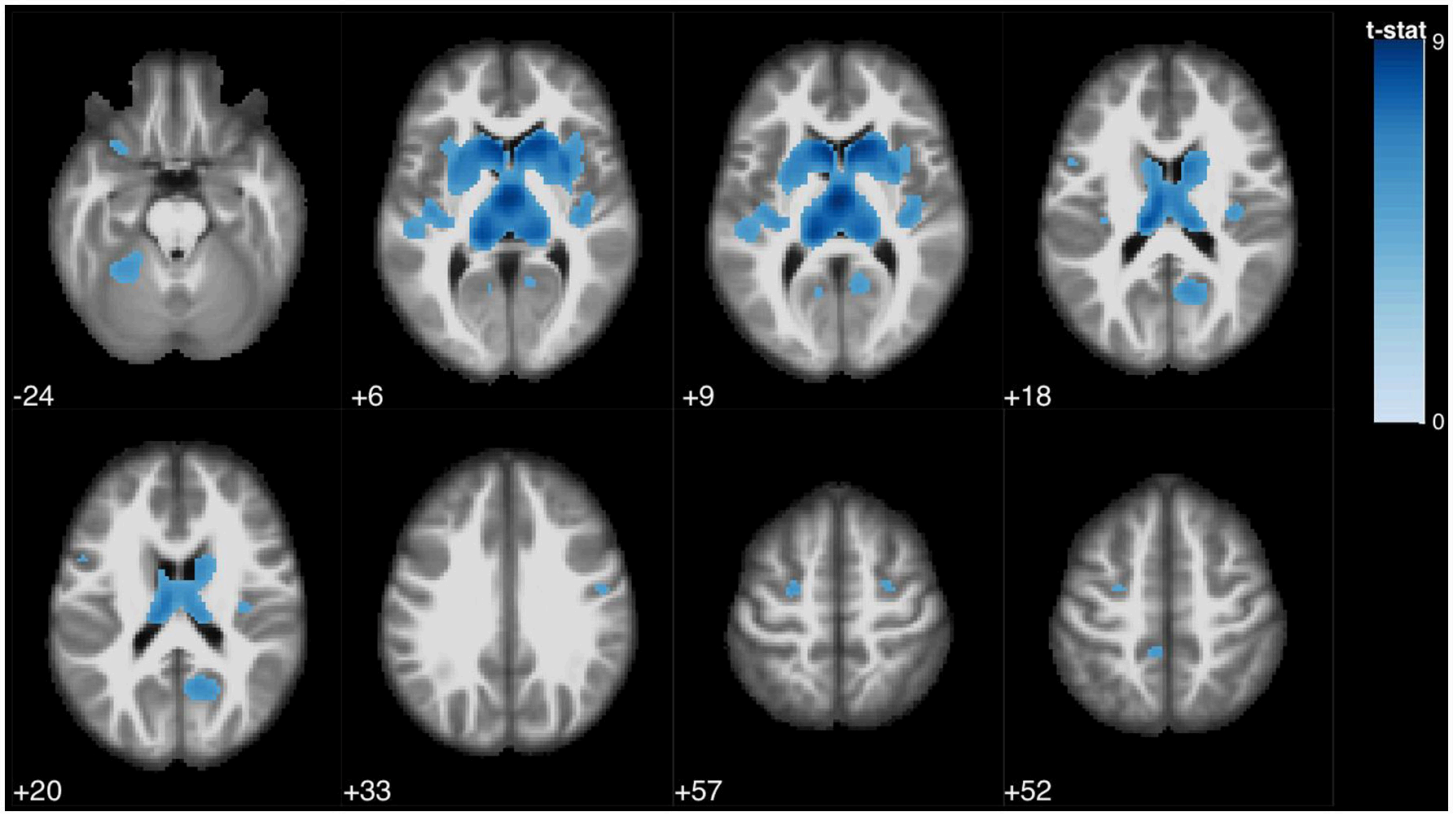
Statistical parametric map overlaid on an MRI template (neurological orientation, axial view), showing brain regions positively correlated with SDMT performance.

**Figure 2:**
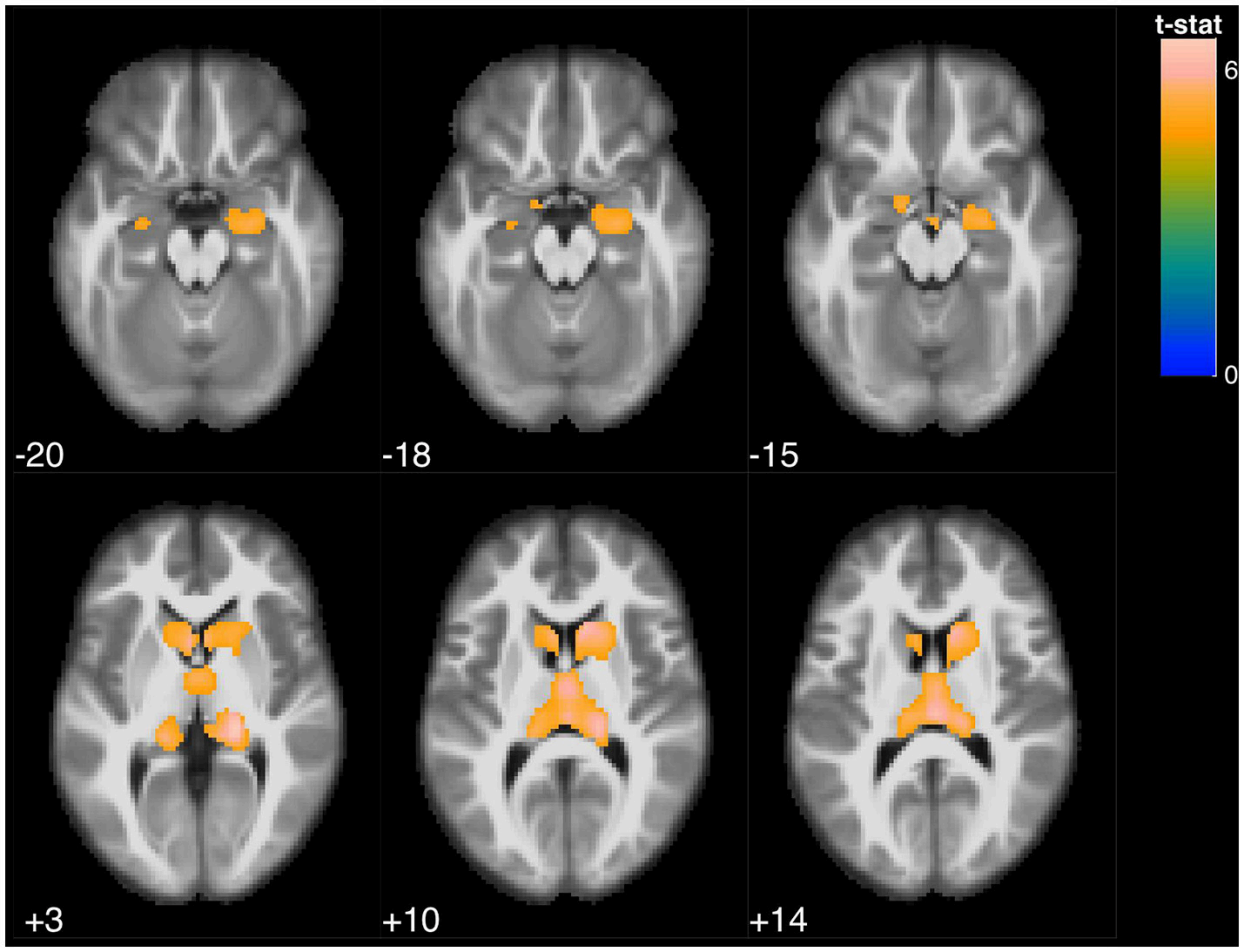
Statistical parametric map overlaid on an MRI template (neurological orientation, axial view), showing brain regions positively correlated with BNT.

**Figure 3:**
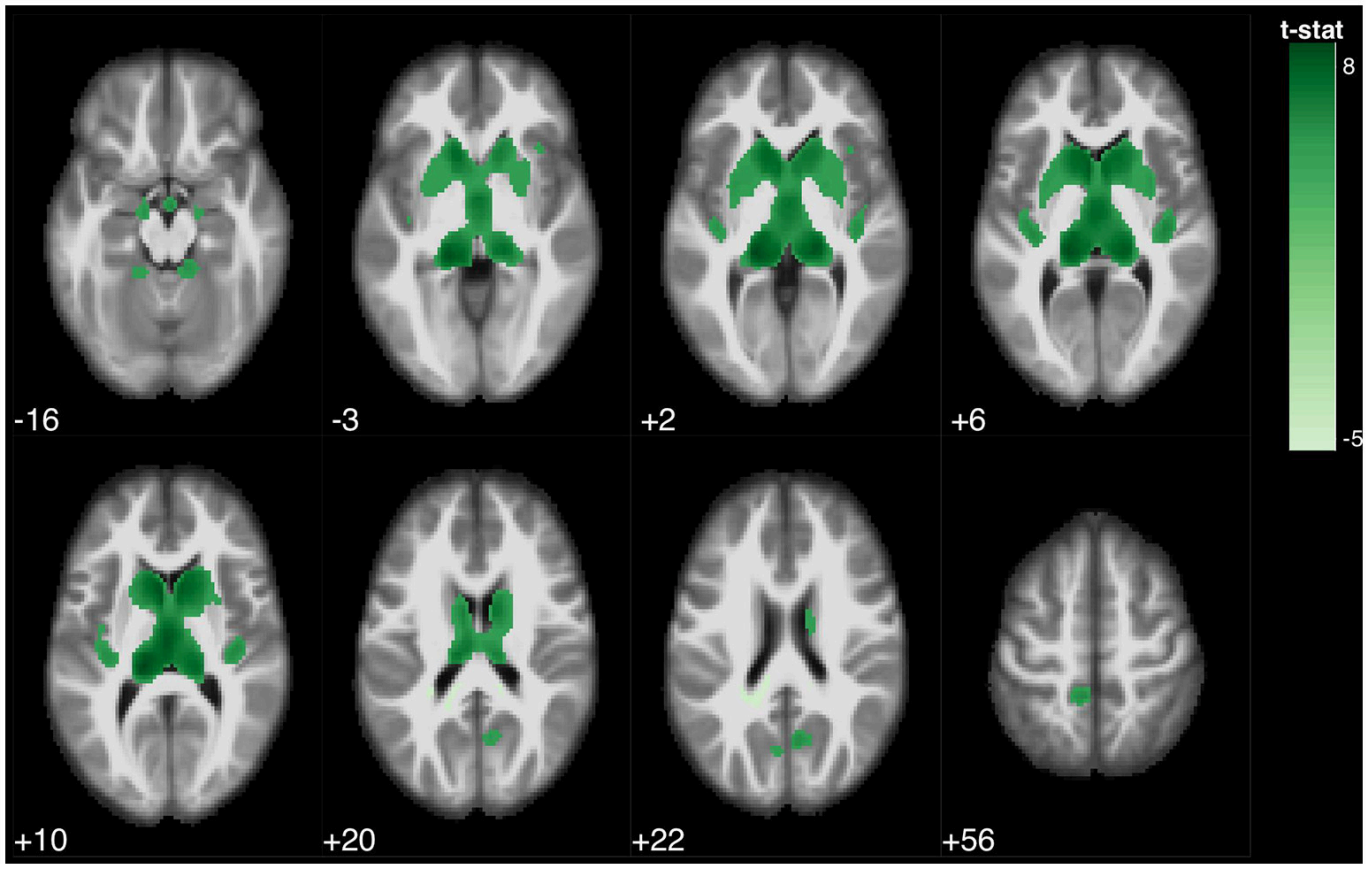
Statistical parametric map overlaid on an MRI template (neurological orientation, axial view), showing brain regions positively correlated with FCSRT (total free recall).

**Figure 4:**
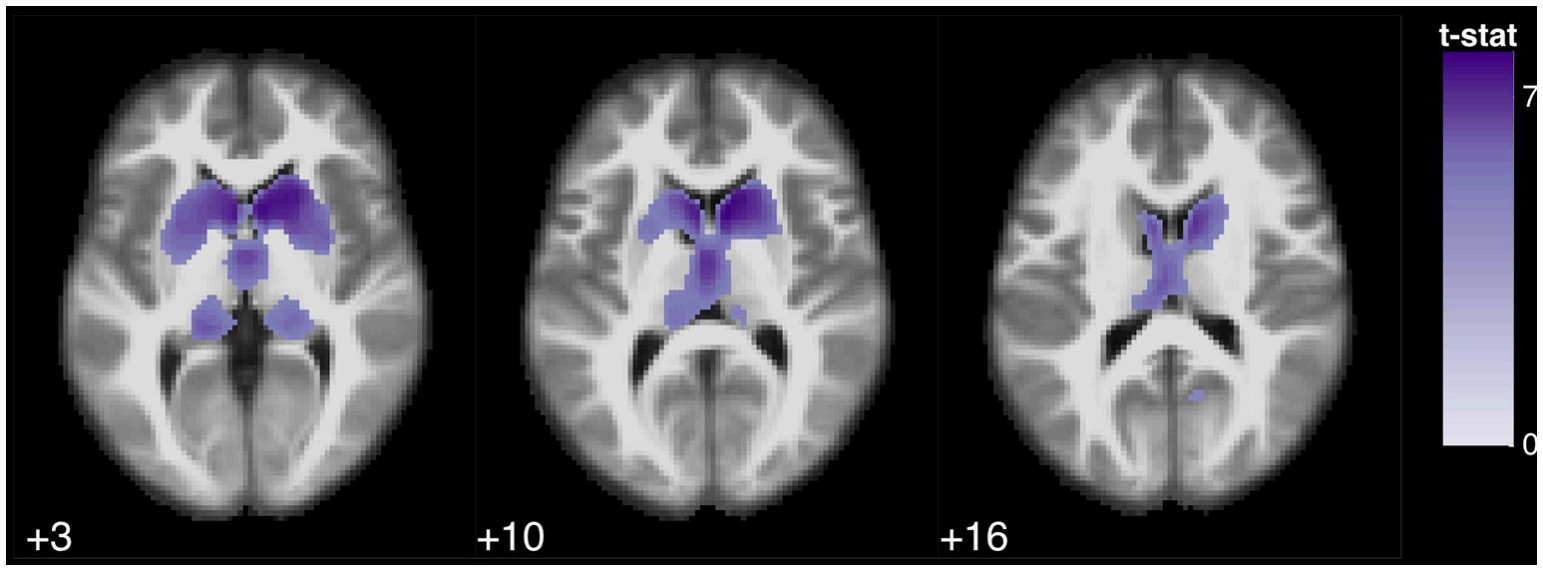
Statistical parametric map overlaid on an MRI template (neurological orientation, axial view), showing brain regions positively correlated with verbal fluencies. Animals, in *purple*.

**Figure 5:**
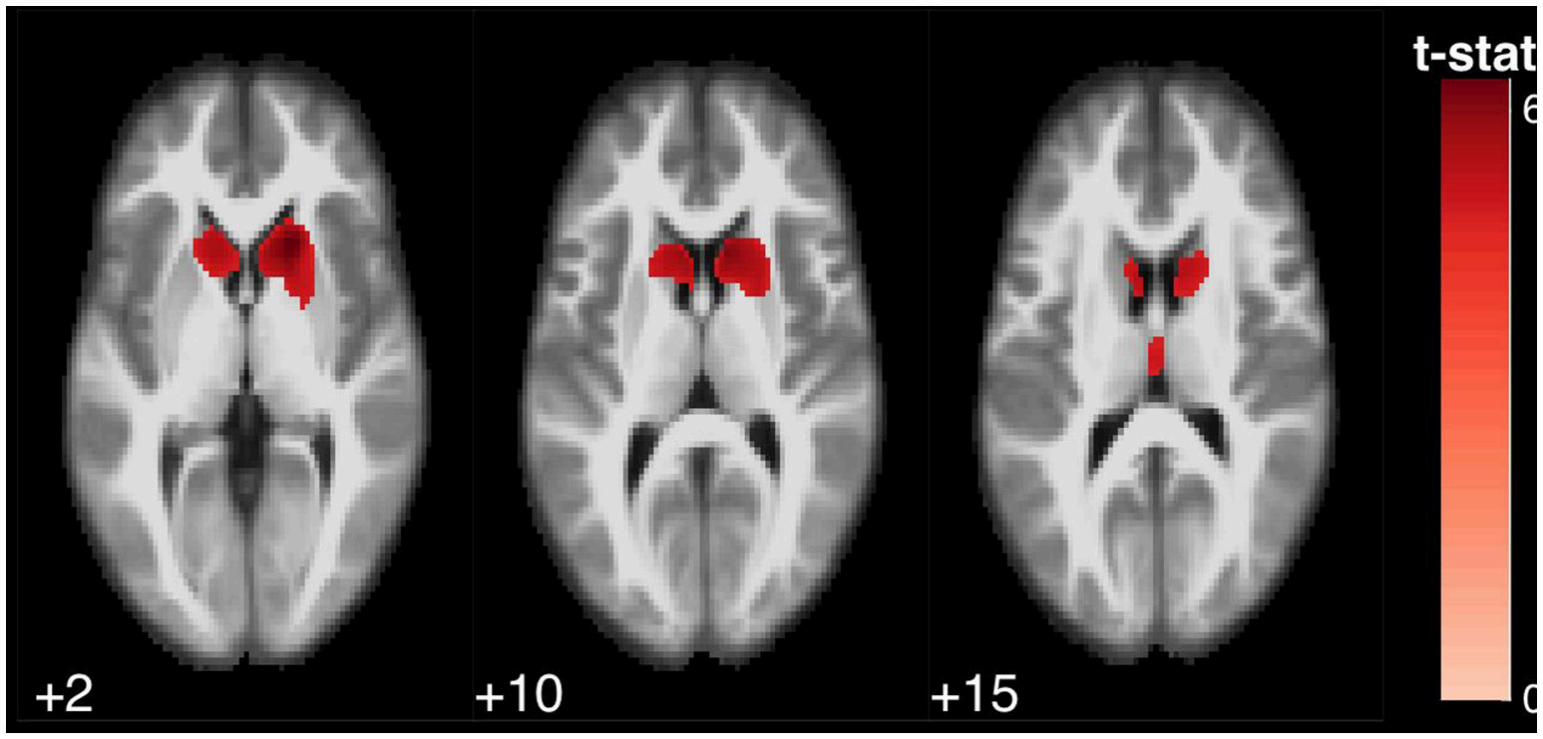
Statistical parametric map overlaid on an MRI template (neurological orientation, axial view), showing brain regions positively correlated with verbal fluencies. “M” words, in *red*.

**Figure 6:**
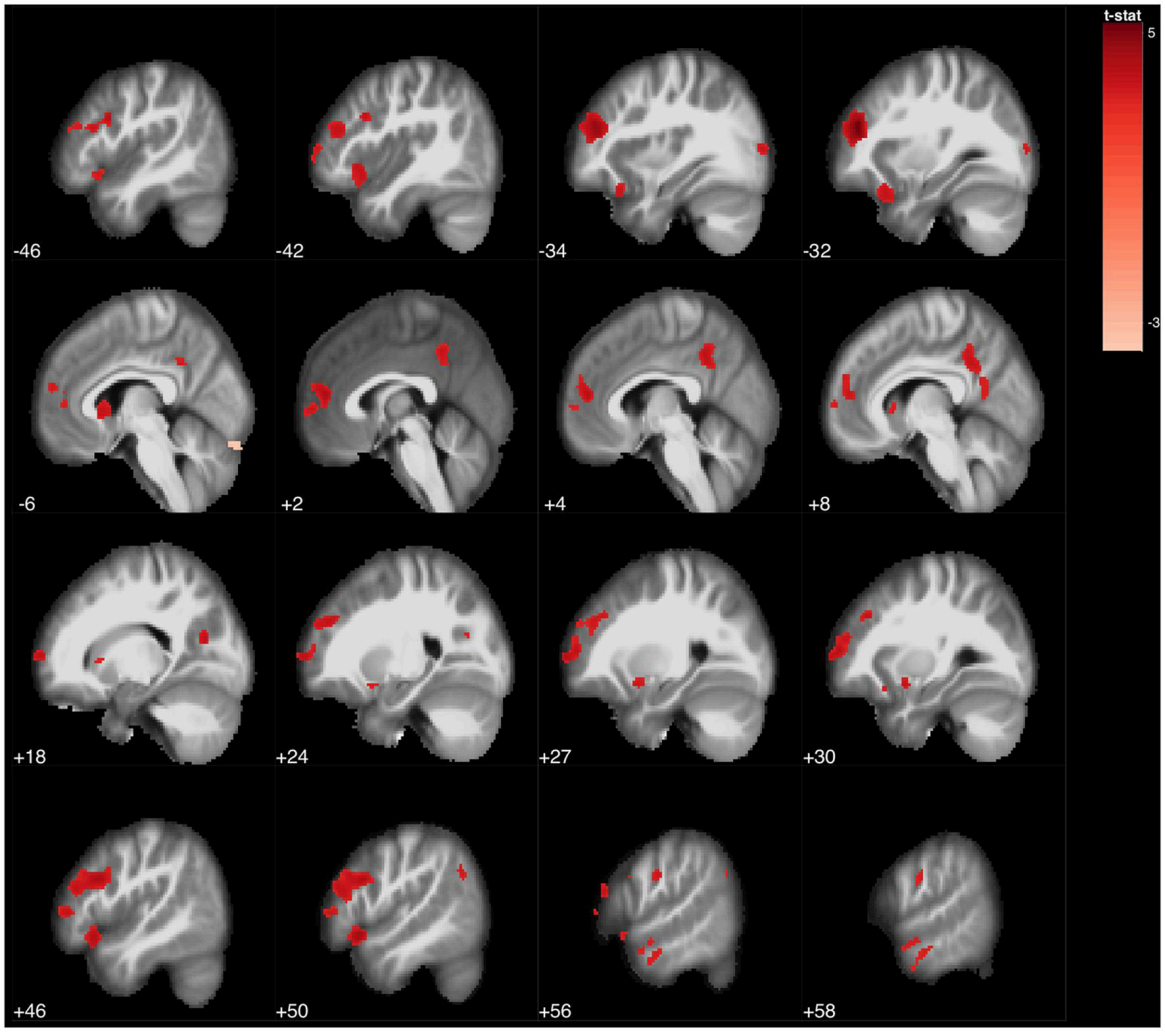
Statistical parametric map overlaid on an MRI template (neurological orientation, sagittal view), showing brain regions positively correlated with Tower of London (uncorrected *p* < 0.001).

We conducted a multiple regression analysis using VBM to identify the brain regions associated with each neuropsychological test. Age, years of formal education, sex, MRI protocol, and total intracranial volume were included in the statistical model as nuisance covariates. Statistical significance was set at *p* < 0.05 using family-wise error (FWE) correction for multiple comparisons at cluster level. A minimum cluster size of *k* = 30 was also used. When non-significant results were found using that threshold in a specific cognitive test, an uncorrected *p* < 0.001 was used to explore brain regions potentially associated with that test. In this case, a minimum cluster size of *k* = 100 was set. The graphical interface working in Matlab® *bspmview* was used for generating Figures 1–6.

## Results

### Main characteristics of patients with and without cognitive impairment

One hundred and sixty-one patients (42.9%) had cognitive impairment. These patients had a lower level of education (14.42 ± 3.90 years of schooling vs. 15.70 ± 3.32, *t* = 3.329, *p* = 0.001), a greater lesion burden (24.16 ± 23.37 mL vs. 12.31 ± 15.32 mL, *t* = −5.590, *p* < 0.0001), a smaller raw gray matter volume (545 ± 67 vs. 563 ± 57, *t* = 2.817, *p* = 0.001), and a smaller normalized gray matter volume (0.405 ± 0.032 vs. 0.419 ± 0.027, *t* = 4.298, *p* < 0.0001). No significant differences were found in age (47.53 ± 10.76 vs. 46.87 ± 9.90, *t* = −0.614, *p* = 0.540) or white matter volume (527 ± 65 vs. 530 ± 65, *t* = 0.424, *p* = 0.672) between patients with and without cognitive impairment. Results of cognitive testing are shown in Table 2.

**Table 2:** Cognitive testing results.

	**Mean (raw scores)**	**Standard deviation (SD)**	**Interquartile range (Q1–Q3)**	**Mean scaled-score ± SD**
Forward digit span	6.01	1.21	5–7	10.02 ± 3.08
Backward digit span	4.27	1.16	3–5	9.18 ± 2.36
Corsi's forward	5.63	0.948	5–6	10.43 ± 2.37
Corsi's backward	4.87	1.02	4–6	9.20 ± 2.78
TMT-A	50.02	30.98	33–56	6.67 ± 3.15
TMT-B	106.21	79.16	65–121	7.10 ± 2.65
SDMT	38.13	14.13	27–48	6.53 ± 2.85
BNT (/60)	52.13	5.26	50–56	9.98 ± 2.94
JLO (/30)	21.37	4.94	18–25	7.89 ± 2.80
ROCF (copy accuracy) (/36)	32.61	5.06	32–36	11.00 ± 4.07
ROCF (memory at 3 min) (/36)	16.19	6.76	11.5–21	8.42 ± 2.90
ROCF (memory at 30 min) (/36)	16.05	6.82	12–21	8.40 ± 2.93
FCSRT (free recall 1) (/16)	9.59	2.48	8–11	12.55 ± 3.43
FCSRT (total free recall) (/48)	31.14	7.71	27–37	10.02 ± 3.72
FCSRT (total recall) (/48)	43.25	6.30	41–47	10.45 ± 4.61
FCSRT (delayed free recall) (/16)	10.65	3.49	8–13	9.43 ± 5.52
FCSRT (delayed total recall) (/48)	14.32	2.47	13-16	11.75 ± 5.78
Verbal fluency (animals)	21.23	6.51	17-26	8.86 ± 6.26
Verbal fluency (“P)	15.25	5.57	11-19	8.61 ± 3.00
Verbal fluency (“M”)	13.19	5.23	10-17	9.06 ± 2.92
Verbal fluency (“R”)	13.05	5.11	10-16	9.48 ± 3, 03
SCWT-A	96.20	21.76	83-110	7.75 ± 3.39
SCWT-B	63.73	14.57	56-74	7.81 ± 3.14
SCWT-C	36.70	12.18	28-45	7.40 ± 2.98
ToL (correct moves) (/10)	4.17	2.30	2-6	9.70 ± 3.15

*Scores are shown as raw and adjusted-scaled scores. Scaled-scores ranges from 2 to 18 (mean 10, standard deviation 3) (e.g., scaled score of 7 is equal to Z-score = – 1)*.

### Correlation with global measures

All tests correlated significantly with lesion load and normalized gray matter volume, except for the forward and backward digit span, ROCF (copy accuracy, recall at 3 and 30 min), and some ToL scores with normalized gray matter volume. Lesion burden was moderately correlated with the SDMT (*r* = −0.462) and the Total Free Recall score in the FCSRT (*r* = −0.449), and only weakly correlated with the remaining tests except for the ROCF (copy accuracy) and ToL (correct moves), the latter with a very weak correlation. Weak correlations were found between normalized gray matter volume and the following tests: TMT-A, TMT-B, SDMT, BNT, ROCF (copy time), FCSRT, ROCF (recognition memory), SCWT (A, B, and C), ToL (total moves), and semantic verbal fluency (animals). Correlations with the remaining tests were very weak. All correlations are shown in Table S1.

Correlations of neuropsychological tests with Beck Depression Inventory were very weak or weak in all cases (Table S2).

### VBM analysis

VBM analysis results and statistics are shown in Table S3. The forward Corsi block-tapping test was associated with a cluster in the right insula, whereas the backward Corsi block-tapping test was associated to the right caudate nucleus and insula. Digit span (forward and backward) showed no suprathreshold clusters.

TMT part A was associated with large clusters in the caudate nucleus and thalamus bilaterally. This test was also correlated with several clusters involving the right posterior cingulate, left inferior frontal gyrus, left, and right hippocampus and parahippocampal gyrus, left superior temporal gyrus, left cuneus, left middle occipital gyrus, bilateral insula, and 3 clusters in the anterior and posterior lobes of the cerebellum. Part B was associated with the bilateral caudate nucleus, bilateral thalamus, bilateral posterior cingulate, and left inferior frontal and middle occipital gyri.

The SDMT was associated with several clusters in the bilateral caudate nucleus, bilateral thalamus, bilateral precuneus and posterior cingulate, left paracentrral lobule, left inferior frontal gyrus and right middle frontal gyrus, bilateral insula, bilateral precentral gyrus, and left anterior cerebellum (Figure 1).

Copy accuracy scores in the ROCF were associated with the right precentral and inferior frontal gyri. After lowering the statistical threshold to *p* < 0.001 (uncorrected), this test was associated with bilateral precentral and inferior frontal gyri, bilateral superior frontal gyrus, right medial frontal gyrus, left inferior parietal lobule, left superior and middle temporal gyri, and left occipital gyrus.

ROCF recall at 3 and 30 min was associated with 2 clusters in the left and right thalami. Recognition memory in the ROCF was also associated with the bilateral thalamus, as well as with 2 clusters in the left caudate, right inferior parietal lobe, and left parahippocampal gyrus.

Regarding the JLO test, no clusters were displayed using the predefined statistical threshold. Using an uncorrected *p* < 0.001, JLO was associated with the bilateral thalamus, bilateral posterior cingulate, bilateral cuneus and precuneus, left angular and supramarginal gyri, bilateral middle temporal gyrus, bilateral inferior frontal gyrus, bilateral caudate nucleus, bilateral insula, left inferior parietal lobule, and left anterior cerebellum.

The BNT was associated with 2 large clusters involving the bilateral thalamus and bilateral caudate, and was also associated with bilateral hippocampus and parahippocampal gyrus (Figure 2).

Regarding the FCSRT, all scores were associated with the bilateral thalamus and bilateral caudate. Furthermore, Free Recall 1 was also associated with regional volume of the bilateral precuneus, left paracentral lobule, and right cerebellum; Total Free Recall scores were also associated with bilateral precuneus, right parahippocampal gyrus, left inferior parietal lobe, left paracentral lobe, and bilateral insula; and Delayed Free Recall scores were also associated the left parahippocampal gyrus, right precuneus, and bilateral insula (Figure 3).

Semantic verbal fluency was associated with a cluster in the bilateral caudate nucleus, extending to the putamina and the bilateral thalamus, and also with a small cluster in the anterior lobe of the cerebellum. Conversely, phonological verbal fluency scores were mainly associated with atrophy of the right and/or left caudate nucleus (Figures 4, 5).

SCWT A was associated mainly with 2 clusters in the right and left caudate nuclei, extending to the putamina. Additional clusters affecting the right cerebellum (posterior lobe), right thalamus, bilateral inferior frontal gyrus, and left supramarginal and superior temporal gyri were also found. SCWT B was associated to bilateral caudate and thalamus and cerebellum. SCWT C was associated to 2 large clusters involving the left and right caudate nuclei and the putamina.

ToL (correct moves) was associated to a small cluster involving the left middle frontal gyrus. Using an uncorrected *p* < 0.001, the ToL test was found to be associated with several regions of the bilateral frontal lobe (superior, middle, medial, and inferior frontal gyrus, and anterior cingulate), bilateral caudate nucleus, and bilateral precuneus and posterior cingulate gyri (Figure 6).

## Discussion

Our study analyses the neural correlates of several cognitive tests in a large cohort of patients with MS who were assessed with MRI and a comprehensive neuropsychological battery. We analyzed global and regional correlations with each test. Nearly all tests showed a significant correlation with white matter lesion burden and normalized gray matter volume. However, correlations were generally weak or very weak. Only SDMT and FCSRT scores exhibited a moderate correlation with white matter lesion burden, suggesting that lesion load may have a more severe impact on memory and information processing speed than on other cognitive functions.

The most interesting finding was the association between the different cognitive tests and regional atrophy. Most tests showed a significant association with atrophy of the thalamus and caudate nucleus. This emphasizes the important role of subcortical structures, and particularly the thalamus and caudate nucleus, in cognitive function in MS. Previous studies have associated thalamic atrophy with general cognitive impairment (23, 24) and information processing speed (17, 44). We hypothesize that subcortical structures play a significant role in all the cognitive functions examined in our study, in view of the association between large clusters within these structures and the results of almost all tests. Intriguingly, although both the thalamus and the caudate nucleus participate in most tests, the caudate nucleus was more strongly associated with tests assessing attention and executive function (e.g., phonological verbal fluency, ToL, etc.) whereas the thalamus was more strongly correlated with tests evaluating memory (e.g., ROCF recall, FCSRT). The putamen was associated with SDMT, SCWT (A, B, and C), and verbal fluency (animals), confirming the role of this structure in information processing speed and time-dependent tasks, as has been suggested (45).

Other regions were also found to be involved in cognitive function. In this regard, we found an association between volumes of the precuneus and posterior cingulate and TMT-A, TMT-B, SDMT, ToL, and FCSRT scores. This region is a key node in the default mode network; it is highly connected with other regions including the thalamus, and it is necessary for highly demanding attentional tasks (46). Our results are consistent with functional MRI studies showing connectivity alterations at this level in patients with cognitive impairment (47). Furthermore, the hippocampus and parahippocampal gyrus were associated with FCSRT (Free Recall), ROCF (memory recognition) and BNT scores. In contrast, visuospatial memory recall measured using ROCF was only associated with the bilateral thalamus and caudate nucleus. Previous studies have reported an association between atrophy of subcortical and medial temporal lobe regions and memory impairment in MS (48, 49). In a large study analysing these regions specifically, Sumowski et al. (49) recently associated memory performance with medial temporal lobe atrophy in relapsing MS and with the thalamus/caudate nucleus in progressive MS. Concerning subcortical regions, both verbal and visuospatial memory tests were more strongly associated with the thalamus than with the caudate nucleus. FCSRT and ROCF (delayed recall and recognition memory) scores were also associated with the caudate nucleus; FCSRT Delayed Free Recall scores were also correlated with the parahippocampal gyrus and precuneus, and ROCF recognition memory scores with the inferior parietal lobule. This confirms the involvement of several structures (mainly the thalamus, caudate nucleus, medial temporal lobe, and precuneus) in the pathophysiology of memory impairment in MS.

The neural correlates of Tower of London test performance constitute another interesting finding of our study. ToL test performance has been correlated with planning and higher-order executive functioning; a previous study using functional MRI in healthy controls and patients with MS has found a correlation between the ToL test and frontal and parietal lobe activation (50). In our sample, a worse performance on this test was associated with bilateral atrophy of several regions of the frontal lobe (including the anterior cingulate and the medial frontal, inferior, middle, and superior frontal gyri) and of the posterior cingulate. Conversely, ToL test scores were not associated with volumes of any subcortical region. These results are similar to those observed in patients with Alzheimer disease, where the Tower of London test has been found to be one of the few executive tests associated specifically with the frontal lobe, in contrast to other more attentional tasks such as the TMT or the SDMT, which were found to be associated with parietal regions linked to attention (51). This suggests a dissociable pattern within the neural correlates of executive functioning: the tests more strongly associated with a high level of attention are linked to several regions involved in attention and, particularly in MS, in subcortical structures; in contrast, the ToL test (which is more closely related to higher-order executive control) was associated with the frontal lobe.

Furthermore, some cerebellar clusters were found to be correlated the SCWT (A, B, and C), verbal fluency (animals), JLO, TMT-A, and SDMT. The importance of the cerebellum in cognitive function (mainly executive function, visuospatial ability, and language) has been emphasized in recent years (52, 53). Some studies have examined the role of cerebellar atrophy in cognitive impairment in MS, usually reporting a correlation with tests assessing information processing speed (54–56). The cognitive tests correlated with cerebellum in our study are all time-dependent, except for the JLO test, and all require precise adjustments to be made during the performance of the test. This may be explained by the influence of information processing speed, as previously suggested (57), and also by the hypothetical role of the cerebellum in performing “online” cognitive adjustments during behavioral tasks, much in the same way as at a motor level (52, 53).

Interestingly, correlations were generally observed bilaterally. Only the Corsi block-tapping test showed a specific correlation with the right hemisphere, confirming the visuospatial nature of the test. This suggests that cognitive assessment in MS should not be interpreted according to the traditional model of left (verbal) vs. right (visuospatial) hemisphere specialization, at least in the context of regional atrophy.

Based on the brain regions associated with each test, we may classify cognitive tests into the following 3 general types: (1) those associated only with subcortical regions (SCWT, phonological verbal fluency); (2) those associated only with cortical regions (ROCF copy accuracy only); and (3) those associated with both cortical and subcortical regions (the remaining tests). Table S4 shows the correlations between different brain regions and neuropsychological tests. Given that most tests were associated with both cortical and subcortical regions, our study supports the idea of a vertical organization of cognitive function. The caudate nucleus and thalamus constitute an essential part of corticostriatal circuits. The caudate nucleus receives input from several cortical structures including the dorsolateral and lateral orbitofrontal cortices, the inferior temporal lobe, and the parietal cortex, while the thalamus receives input from the basal ganglia and projects to the cortex (57). According to this hypothesis of a vertical organization of the brain, subcortical structures (the basal ganglia and cerebellum) modulate cognitive function in association with the cortex, as occurs in motor control (57).

Our study has some limitations. The time between the performance of the MRI scan and the neuropsychological assessment varied; this period was of significant length in some cases. We attempted to minimize the effect of this potential limitation by excluding all patients with clinical relapses. Furthermore, MRI scans were performed on a 1.5 T scan with two different 3D-T1-weighted protocols regarding slice thickness with 3 or 1 mm. We tried to reduce this limitation adding the MRI protocol to the statistical model as a covariate. In this regard, other potential confounding factors were added as covariates in the multiple regression analysis (sex, age, years of education, TICV). We did not include the scoring in the Beck Depression Inventory, due to the weak correlations with neuropsychological tests. Although depression has been previously associated to poorer cognitive performance in MS (58), the influence in the neuropsychological protocol used in our study was low, explaining a low percentage of the cognitive scores. Furthermore, in a previous study we conducted to identify the principal components of our neuropsychological battery, depression constituted a separate cluster from cognitive tests, with no significant impact on the cognitive domains in MS (2). Moreover, our analyses were focused on lesion load, global brain volume, and VBM, which has their own limitations (59). Other MRI analyses, sequences and techniques, such as surface-based cortical thickness, diffusion tensor imaging or high-field MRI for cortical lesions, should be used to confirm our findings and evaluate other aspects of MS pathophysiology that may help to explain the neural basis of cognitive function. These types of studies improve the interpretation of cognitive assessment findings and assist in the selection of the most suitable neuropsychological tests for a specific disease by examining a large number of brain functions, networks, and regions (51). Furthermore, our study has a cross-sectional design; longitudinal studies are necessary to establish the sequence of events resulting in cognitive impairment and the specific roles of different regions. In addition, we did not include a healthy control group, which may be interesting in order to elucidate potential differences in the associations between gray matter volumes and cognitive performance between MS patients and healthy controls.

In conclusion, our study examines the neural correlates of specific neuropsychological tests in MS using VBM. Within subcortical structures, the neuropsychological tests primarily assessing attention and executive function were more strongly associated with caudate volumes, while those mainly evaluating memory were more strongly associated with the thalamus. Other relevant regions included the posterior cingulate/precuneus, associated with attentional tasks, and several frontal regions, associated with planning. Our results improve the current understanding of the neural basis of cognitive dysfunction in MS, and specifically of each of the cognitive domains. Furthermore, this study provides additional data about the role of cortical and subcortical regions in the pathophysiology of cognitive impairment in MS, drawing attention to the vertical organization of cognitive function, involving the cortex, thalamus, basal ganglia, and cerebellum. Given the high frequency of cognitive dysfunction in MS and the need to define biomarkers of disease stability and/or progression, establishing the brain areas associated with cognitive alterations may open new pathways defining potential biomarkers.

## Ethical approval

This study was conducted with the approval of our hospital's Ethics Committee.

## Research involving human participants

All procedures performed were in accordance with the ethical standards of the institutional research committee and with the 1964 Helsinki declaration and its later amendments.

## Informed consent

Written informed consent was obtained from all individual participants included in the study.

## Availability of data and material

The datasets used and/or analyzed during the current study are available from the corresponding author on reasonable request.

## Ethics statement

This study was carried out in accordance with the recommendations of name of guidelines, name of committee with written informed consent from all subjects. All subjects gave written informed consent in accordance with the Declaration of Helsinki. The protocol was approved by the Ethics Research Committee from the Hospital Clinico San Carlos.

## Author contributions

JAM-G design of the study, statistical analysis, interpretation of data, writing of the manuscript, and final approval of the manuscript. AC-M data acquisition, statistical analysis, literature review, interpretation of data, writing of the manuscript, and final approval of the manuscript. PM data acquisition, literature review, interpretation of data, and final approval of the manuscript. VP data acquisition, design of the study, and final approval of the manuscript. TM-R data acquisition, literature review, and final approval of the manuscript. MY data acquisition, study supervision, critical revision of manuscript for important intellectual content, and final approval of the manuscript. MJ and TM-R data acquisition, literature review, and final approval of the manuscript. JA design of the study, data acquisition, and final approval of the manuscript. JM-G design of the study, study supervision, interpretation of data, critical revision of manuscript for important intellectual content, and final approval of the manuscript.

### Conflict of interest statement

The authors declare that the research was conducted in the absence of any commercial or financial relationships that could be construed as a potential conflict of interest.
