# Supplementary material for: Identification of Cortical and Subcortical Correlates of Cognitive Performance in Multiple Sclerosis Using Voxel-Based Morphometry
Source: Front Neurol. 2018 Oct 29;9:920. doi: 10.3389/fneur.2018.00920 (PMC6216547; doi:10.3389/fneur.2018.00920)
Supplement: Supplementary file 1 [file Table_1.DOCX]

| Table S1. Correlations between cognitive tests and lesion load and normalized gray matter volumen.  *Numbers in cells represent r coefficient (p-value).*  *Significant values after Bonferroni correction are shown in bold.*  *Very weak correlations are marked with gray, weak correlation with yellow, and moderate correlations with orange.* | | |
| --- | --- | --- |
|  | ***Lesion load*** | ***Normalized gray matter volume*** |
| Forward digit span | -0.164 (0.00149) | 0.104 (0.045) |
| Backward digit span | **-0.224 (<0.0001)** | 0.089 (0.088) |
| Corsi’s block forward | **-0.201 (<0.0001)** | 0.139 (0.007) |
| Corsi’s block backward | **-0.257 (<0.0001)** | 0.176 (0.0006) |
| TMT-A | **0.394 (<0.0001)** | **-0.348 (<0.0001)** |
| TMT-B | **0.372 (<0.0001)** | **-0.290 (<0.0001)** |
| SDMT | **-0.462 (<0.0001)** | **0.345 (<0.0001)** |
| Boston Naming Test | **-0.336 (<0.0001)** | **0.230 (<0.0001)** |
| ROCF (copy accuracy) | **-0.168 (0.001)** | 0.155 (0.003) |
| ROCF (copy time) | **0.273 (<0.0001)** | **-0.209 (<0.0001)** |
| Judgement Line Orientation | **-0.266 (<0.0001)** | 0.138 (0.009) |
| FCSRT-Free Recall 1 | **-0.368 (<0.0001)** | **0.252 (<0.0001)** |
| FCSRT-Total Free Recall | **-0.449 (<0.0001)** | **0.282 (<0.0001)** |
| FCSRT-Total Recall | **-0.346 (<0.0001)** | **0.185 (<0.0001)** |
| FCSRT-Delayed Free Recall | **-0.365 (<0.0001)** | **0.256 (<0.0001)** |
| FCSRT-Delayed Total Recall | **-0.294 (<0.0001)** | **0.152 (<0.0001)** |
| ROCF (memory at 3 minutes) | **-0.278 (<0.0001)** | 0.154 (0.004) |
| ROCF (memory at 30 minutes) | **-0.299 (<0.0001)** | **0.186 (<0.0001)** |
| ROCF (memory – recognition) | **-0.324 (<0.0001)** | **0.221 (<0.0001)** |
| Stroop A | **-0.290 (<0.0001)** | **0.223 (<0.0001)** |
| Stroop B | **-0.306 (<0.0001)** | **0.220 (<0.0001)** |
| Stroop C | **-0.300 (<0.0001)** | **0.204 (<0.0001)** |
| ToL (correct moves score) | **-0.194 (<0.0001)** | 0.131 (0.014) |
| ToL (total moves score) | **0.306 (<0.0001)** | **-0.170 (0.0013)** |
| ToL (initiation time) | 0.085 (0.118) | -0.051 (0.351) |
| ToL (execution time) | **0.225 (<0.0001)** | -0.128 (0.018) |
| ToL (problem-solving time) | **0.301 (<0.0001)** | **-0.1575 (0.0012)** |
| Verbal fluency (animals) | **-0.341 (<0.0001)** | **0.260 (<0.0001)** |
| Verbal fluency (“p” words) | **-0.294 (<0.0001)** | **0.179 (0.0005)** |
| Verbal fluency (“m” words) | **-0.281 (<0.0001)** | **0.177 (0.0006)** |
| Verbal fluency (“r” words) | **-0.274 (<0.0001)** | **0.179 (0.0005)** |
