# Supplementary material for: Identification of Cortical and Subcortical Correlates of Cognitive Performance in Multiple Sclerosis Using Voxel-Based Morphometry
Source: Front Neurol. 2018 Oct 29;9:920. doi: 10.3389/fneur.2018.00920 (PMC6216547; doi:10.3389/fneur.2018.00920)
Supplement: Supplementary file 2 [file Table_2.DOCX]

| **Table S2.**  **Pearson’s correlation coefficient between cognitive tests and Beck Depression Inventory** | |
| --- | --- |
| *Neuropsychological test* | *Rho coefficient (p-value)* |
| Forward digit span | -0.152 (0.003) |
| Backward digit span | -0.142 (0.006) |
| Corsi’s block forward | -0.129 (0.013) |
| Corsi’s block backward | -0.168 (0.001) |
| TMT-A | 0.113 (0.032) |
| TMT-B | 0.059 (0.271) |
| SDMT | -0.156 (0.003) |
| Boston Naming Test | -0.100 (0.053) |
| ROCF (copy accuracy) | -0.147 (0.006) |
| ROCF (copy time) | 0.060 (0.255) |
| Judgement Line Orientation | -0.079 (0.138) |
| FCSRT-Free Recall 1 | -0.129 (0.013) |
| FCSRT-Total Free Recall | -0.133 (0.010) |
| FCSRT-Total Recall | -0.128 (0.014) |
| FCSRT-Delayed Free Recall | -0.198 (<0.0001) |
| FCSRT-Delayed Total Recall | -0.236 (<0.0001) |
| ROCF (memory at 3 minutes) | -0.118 (0.026) |
| ROCF (memory at 30 minutes) | -0.130 (0.014) |
| ROCF (memory – recognition) | -0.095 (0.073) |
| Stroop A | -0.269 (<0.0001) |
| Stroop B | -0.226 (<0.0001) |
| Stroop C | -0.238 (<0.0001) |
| ToL (correct moves score) | -0.002 (0.963) |
| Verbal fluency (animals) | -0.190 (<0.0001) |
| Verbal fluency (“p” words) | -0.191 (<0.0001) |
| Verbal fluency (“m” words) | -0.132 (0.011) |
| Verbal fluency (“r” words) | -0.199 (<0.0001) |
