# Supplementary material for: Identification of Cortical and Subcortical Correlates of Cognitive Performance in Multiple Sclerosis Using Voxel-Based Morphometry
Source: Front Neurol. 2018 Oct 29;9:920. doi: 10.3389/fneur.2018.00920 (PMC6216547; doi:10.3389/fneur.2018.00920)
Supplement: Supplementary file 3 [file Table_3.docx]

| **Table S3.** Voxel-based morphometry analysis. Multiple regression analysis showing correlations between cognitive tests and brain regions, using age, years of education, sex, MRI protocol, and total intracranial volume as covariates  FWE corrected p-value <0.05, k=30.  (*): Negative correlations.  (&): Uncorrected p-value <0.001, k=100. | | | | | | | | | | | | |
| --- | --- | --- | --- | --- | --- | --- | --- | --- | --- | --- | --- | --- |
| Brain region (Brodmann area) | | MNI coordinates | | | | | T value | | Z score | | K (number of voxels) | |
|  |  | x | | y | | z |  |  |  |  |  |  |
| *Regions correlated with Corsi block-tapping test (forward)* | | | | | | | | | | | | |
| Right insula | | 26 | | 18 | | 7 | 5.35 | | 5.25 | | 167 | |
|  |  | 39 | | 20 | | 4 | 5.18 | | 5.09 | |  |  |
| *Regions correlated with Corsi block-tapping test (backward)* | | | | | | | | | | | | |
| Right caudate  and right insula | | 26 | | 18 | | 7 | 5.35 | | 5.25 | | 167 | |
|  |  | 39 | | 20 | | 4 | 5.18 | | 5.09 | |  |  |
| *Regions correlated with Trail Making Test part A** | | | | | | | | | | | | |
| Left caudate and bilateral thalamus | | –10 | | 15 | | –2 | 7.06 | | 6.82 | | 7352 | |
|  |  | 16 | | -30 | | 7 | 6.49 | | 6.30 | |  |  |
|  |  | –14 | | -28 | | 12 | 6.20 | | 6.03 | |  |  |
| Right caudate | | 9 | | 15 | | 10 | 6.99 | | 6.76 | | 2781 | |
|  |  | 15 | | 15 | | 1 | 6.97 | | 6.74 | |  |  |
| Left cerebellum (anterior lobe, culmen) | | –28 | | –49 | | -24 | 6.37 | | 6.20 | | 1690 | |
|  |  | -39 | | -45 | | -29 | 5.97 | | 5.82 | |  |  |
| Left middle occipital gyrus (18, 19) | | -33 | | -90 | | 9 | 6.20 | | 6.04 | | 560 | |
|  |  | -30 | | -91 | | 1 | 5.97 | | 5.82 | |  |  |
| Right insula (13) | | 39 | | -19 | | 7 | 6.13 | | 5.97 | | 784 | |
| Right posterior cingulate (31) | | 12 | | -58 | | 16 | 6.04 | | 5.89 | | 1227 | |
| Left insula and left superior temporal gyrus (13, 22) | | -42 | | -18 | | 4 | 5.99 | | 5.84 | | 717 | |
|  |  | -52 | | -27 | | 6 | 5.43 | | 5.32 | |  |  |
| Left cuneus (19) | | -28 | | -82 | | 30 | 5.77 | | 5.64 | | 166 | |
| Right precentral gyrus (6) | | 30 | | -9 | | 57 | 5.75 | | 5.62 | | 271 | |
| Left parahippocampal gyrus and hippocampus (34) | | -15 | | -9 | | -18 | 5.71 | | 5.58 | | 577 | |
|  |  | -27 | | -10 | | -20 | 5.70 | | 5.57 | |  |  |
| Left middle frontal gyrus (6) | | -24 | | -9 | | 58 | 5.71 | | 5.58 | | 101 | |
| Right cingulate gyrus (31) | | 14 | | -34 | | 43 | 5.70 | | 5.57 | | 474 | |
| Left inferior frontal gyrus (47) | | 39 | | 21 | | -5 | 5.56 | | 5.44 | | 219 | |
| Right cerebellum (anterior lobe, culmen) | | 8 | | -40 | | -14 | 5.50 | | 5.39 | | 250 | |
|  |  | 18 | | -39 | | -15 | 5.01 | | 4.92 | |  |  |
| Right para-hippocampal gyrus (28) | | 18 | | -10 | | -20 | 5.41 | | 5.29 | | 316 | |
| Right cerebellum (posterior lobe, inferior) semi-lunar lobule) | | 3 | | -69 | | -41 | 5.34 | | 5.24 | | 144 | |
| Left middle occipital and middle temporal gyri (19, 39) | | -48 | | -75 | | 3 | 5.30 | | 5.19 | | 360 | |
|  |  | -56 | | -66 | | 12 | 5.10 | | 5.01 | |  |  |
| Right cerebellum (anterior lobe, culmen) | | 26 | | -51 | | -24 | 5.12 | | 5.02 | | 105 | |
| *Regions correlated with Trail Making Test part B** | | | | | | | | | | | | |
| Right caudate | | 21 | | 16 | | 7 | 6.74 | | 6.53 | | 2545 | |
|  |  | 9 | | 15 | | 10 | 6.52 | | 6.33 | |  |  |
| Right posterior cingulate (23) | | 9 | | -58 | | 12 | 5.92 | | 5.77 | | 783 | |
| Left caudate | | –12 | | 15 | | –5 | 5.91 | | 5.76 | | 948 | |
|  |  | –4 | | 14 | | 1 | 5.76 | | 5.63 | |  |  |
| Right and left thalamus | | 4 | | –4 | | –3 | 5.84 | | 5.70 | | 2191 | |
|  |  | -15 | | -30 | | 10 | 5.66 | | 5.53 | |  |  |
|  |  | 2 | | –10 | | 10 | 5.48 | | 5.36 | |  |  |
| Left middle occipital gyrus (18) | | –40 | | –87 | | 7 | 5.37 | | 5.26 | | 135 | |
| Left posterior cingulate (30) | | -10 | | -57 | | 7 | 5.22 | | 5.12 | | 102 | |
| Left inferior frontal gyrus (47) | | –34 | | 20 | | –17 | 5.04 | | 4.95 | | 30 | |
| *Regions correlated with Symbol Digit Modalities Test* | | | | | | | | | | | | |
| Right and left caudate, right and left thalamus, right and left putamen | | 9 | | 15 | | 10 | 9.05 | | Inf. | | 22862 | |
|  |  | –6 | | 14 | | 10 | 9.02 | | Inf. | |  |  |
|  |  | 0 | | -12 | | 9 | 9.01 | | Inf. | |  |  |
| Right insula | | 39 | | -19 | | 9 | 6.42 | | 6.24 | | 827 | |
| Left insula and superior temporal gyrus (13, 41) | | -40 | | -18 | | 6 | 6.41 | | 6.23 | | 1114 | |
|  |  | -51 | | -28 | | 9 | 5.73 | | 5.60 | |  |  |
|  |  | -40 | | -7 | | -8 | 5.12 | | 5.03 | |  |  |
| Right precuneus and posterior cingulate (30, 31) | | 12 | | –61 | | 19 | 6.34 | | 6.17 | | 1026 | |
| Left cerebellum (anterior lobe, culmen) | | -28 | | -48 | | -24 | 5.91 | | 5.77 | | 762 | |
|  |  | -18 | | -45 | | -14 | 5.32 | | 5.21 | |  |  |
| Left middle frontal gyrus (6) | | -26 | | -7 | | 57 | 5.78 | | 5.65 | | 122 | |
| Left paracentral lobule (5) | | -6 | | -42 | | 52 | 5.50 | | 5.38 | | 92 | |
| Right precentral gyrus (6) | | 51 | | -7 | | 33 | 5.41 | | 5.30 | | 89 | |
| Right middle frontal gyrus (6) | | 27 | | -6 | | 58 | 5.41 | | 5.30 | | 80 | |
| Left precentral gyrus (4) | | -45 | | -15 | | 42 | 5.38 | | 5.27 | | 63 | |
| Left inferior frontal gyrus (44) | | -52 | | 9 | | 18 | 5.27 | | 5.17 | | 65 | |
| Left posterior cingulate gyrus and precuneus (30) | | -10 | | -63 | | 10 | 5.06 | | 4.97 | | 54 | |
| Right precentral gyrus (4) | | 40 | | -13 | | 45 | 5.04 | | 4.95 | | 36 | |
| *Regions correlated with Stroop Color and Word Test, part A* | | | | | | | | | | | | |
| Right caudate and putamen | | 9 | | 14 | | 10 | 5.96 | | 5.81 | | 1787 | |
|  |  | 32 | | 4 | | 4 | 5.51 | | 5.39 | |  |  |
|  |  | 26 | | 18 | | 6 | 5.50 | | 5.38 | |  |  |
| Left inferior frontal gyrus (9) | | -52 | | 9 | | 24 | 5.54 | | 5.42 | | 121 | |
| Right thalamus | | 3 | | -3 | | -6 | 5.51 | | 5.39 | | 182 | |
| Left supramarginal gyrus (40) | | -50 | | -48 | | 28 | 5.51 | | 5.39 | | 86 | |
|  |  | -52 | | -55 | | 30 | 5.03 | | 4.94 | |  |  |
| Left caudate and putamen | | –8 | | 18 | | 3 | 5.60 | | 5.47 | | 502 | |
| Left superior temporal gyrus (41) | | -52 | | -28 | | 9 | 5.20 | | 5.10 | | 123 | |
| Left putamen | | –26 | | 8 | | 7 | 5.16 | | 5.06 | | 176 | |
| Right frontal gyrus (47) and insula | | 44 | | 18 | | -3 | 5.12 | | 5.02 | | 91 | |
|  |  | 36 | | 21 | | 3 | 5.05 | | 4.96 | |  |  |
| *Regions correlated with Stroop Color and Word Test, part B* | | | | | | | | | | | | |
| Right caudate and putamen | | 9 | | 14 | | 12 | 6.51 | | 6.32 | | 2638 | |
|  |  | 12 | | 18 | | 3 | 6.19 | | 6.03 | |  |  |
|  |  | 30 | | 4 | | 3 | 5.55 | | 5.43 | |  |  |
| Left caudate and putamen | | –4 | | 15 | | 3 | 5.93 | | 5.78 | | 846 | |
|  |  | –9 | | 16 | | –5 | 5.75 | | 5.62 | |  |  |
| Right thalamus | | 3 | | –3 | | -6 | 5.61 | | 5.49 | | 247 | |
| Right cerebellum (inferior semi-lunar lobule, posterior lobe) | | 4 | | –69 | | –41 | 5.57 | | 5.45 | | 266 | |
| Right thalamus | | 2 | | -15 | | 13 | 5.10 | | 5.01 | | 126 | |
| *Regions correlated with Stroop Color and Word Test, part C* | | | | | | | | | | | | |
| Right caudate, right putamen | | 10 | | 14 | | 13 | 6.02 | | 5.87 | | 2447 | |
|  |  | 26 | | 16 | | 6 | 5.74 | | 5.60 | |  |  |
|  |  | 30 | | 9 | | 3 | 5.69 | | 5.56 | |  |  |
| Left caudate | | –9 | | 20 | | 3 | 5.63 | | 5.50 | | 633 | |
| Right thalamus | | 3 | | –4 | | –8 | 5.37 | | 5.27 | | 214 | |
| Left putamen | | –26 | | 9 | | 4 | 5.38 | | 5.27 | | 617 | |
| *Regions correlated with Tower of London (correct moves)* | | | | | | | | | | | | |
| Left middle frontal gyrus (10) | -32 | | 45 | | 22 | | | 5.28 | | 5.17 | | 67 |
| *Regions correlated with Tower of London (correct moves) (&)* | | | | | | | | | | | | |
| Left middle frontal gyrus (9, 10) | | –32 | | 44 | | 22 | 5.28 | | 5.17 | | 1631 | |
|  |  | –27 | | 33 | | 33 | 3.43 | | 3.40 | |  |  |
| Right inferior frontal gyrus (47) | | 50 | | 21 | | -12 | 4.55 | | 4.49 | | 678 | |
|  |  | 34 | | 28 | | -14 | 3.39 | | 3.36 | |  |  |
| Right middle frontal gyrus (9, 10, 46) | | 46 | | 21 | | 28 | 4.32 | | 4.26 | | 3104 | |
|  |  | 40 | | 39 | | 22 | 4.32 | | 4.26 | |  |  |
|  |  | 27 | | 68 | | 6 | 4.00 | | 3.95 | |  |  |
| Right and left medial frontal gyrus (10), left anterior cingulate (32) | | 2 | | 50 | | 13 | 4.21 | | 4.15 | | 814 | |
|  |  | –2 | | 57 | | 18 | 3.93 | | 3.88 | |  |  |
|  |  | -2 | | 50 | | 4 | 3.81 | | 3.77 | |  |  |
| Left inferior and middle frontal gyri (9, 46) | | –46 | | 12 | | 28 | 3.89 | | 3.85 | | 246 | |
|  |  | -46 | | 24 | | 22 | 3.65 | | 3.62 | |  |  |
| Right precuneus (31) | | 18 | | -67 | | 19 | 3.82 | | 3.78 | | 170 | |
| Right and left cingulate gyrus, left precuneus (31) | | 4 | | –46 | | 42 | 3.79 | | 3.75 | | 790 | |
|  |  | 9 | | -51 | | 34 | 3.72 | | 3.68 | |  |  |
|  |  | -8 | | -48 | | 34 | 3.35 | | 3.32 | |  |  |
| Left inferior frontal and superior temporal gyrus (38, 47) | | -42 | | 21 | | -8 | 3.79 | | 3.74 | | 744 | |
|  |  | -32 | | 23 | | -26 | 3.68 | | 3.65 | |  |  |
| Right inferior frontal gyrus (46) | | 46 | | 45 | | 4 | 3.72 | | 3.68 | | 209 | |
| Left caudate | | -6 | | 18 | | 0 | 3.70 | | 3.66 | | 267 | |
| Right para-hippocampal gyrus (34) | | 30 | | 6 | | -14 | 3.64 | | 3.60 | | 145 | |
| Left middle frontal gyrus (10) | | -40 | | 56 | | 7 | 3.62 | | 3.58 | | 133 | |
| Right angular gyrus (39) | | 51 | | -63 | | 33 | 3.60 | | 3.57 | | 105 | |
| Left middle occipital gyrus (19) | | -34 | | -93 | | 7 | 3.58 | | 3.55 | | 105 | |
| Right precentral gyrus (6) | | 56 | | -7 | | 31 | 3.49 | | 3.46 | | 102 | |
| Right precentral and middle temporal gyri (21) | | 58 | | -3 | | 15 | 3.36 | | 3.33 | | 136 | |
|  |  | 58 | | 4 | | -20 | 3.30 | | 3.28 | |  |  |
| Right fusiform and inferior temporal gyri (20) | | 56 | | -6 | | -26 | 3.33 | | 3.30 | | 166 | |
|  |  | 60 | | -15 | | -21 | 3.27 | | 3.24 | |  |  |
| Right posterior cingulate (30) | | 8 | | -60 | | 13 | 3.30 | | 3.27 | | 120 | |
| Right caudate | | 8 | | 15 | | 3 | 3.27 | | 3.25 | | 157 | |
|  |  | 18 | | 21 | | 3 | 3.18 | | 3.16 | |  |  |
| *Regions correlated with Free and Cued Selective Reminding Test (free recall 1)* | | | | | | | | | | | | |
| Left and right thalamus | | 15 | | –31 | | 1 | 6.88 | | 6.67 | | 5637 | |
|  |  | -18 | | -31 | | 1 | 6.70 | | 6.50 | |  |  |
|  |  | 0 | | -16 | | 12 | 6.66 | | 6.46 | |  |  |
| Right caudate | | 9 | | 10 | | 12 | 6.72 | | 6.52 | | 1515 | |
| Left caudate | | –4 | | 10 | | 7 | 6.30 | | 6.13 | | 1226 | |
|  |  | -9 | | 18 | | 3 | 6.20 | | 6.04 | |  |  |
| Left precuneus (7) and left paracentral lobule (5) | | –6 | | –43 | | 54 | 5.71 | | 5.58 | | 149 | |
|  |  | –3 | | –48 | | 46 | 5.01 | | 4.92 | |  |  |
| Right precuneus (31) | | 8 | | –66 | | 24 | 5.51 | | 5.39 | | 151 | |
| Right cerebellum (anterior lobe, culmen) | | 10 | | –39 | | -15 | 5.41 | | 5.30 | | 105 | |
| *Regions correlated with Free and Cued Selective Reminding Test (total free recall)* | | | | | | | | | | | | |
| Left and right thalamus, left and right caudate | | –15 | | -28 | | 10 | 8.84 | | Inf | | 17957 | |
|  |  | -18 | | -31 | | 0 | 8.78 | | Inf | |  |  |
|  |  | -2 | | -18 | | 13 | 8.64 | | Inf | |  |  |
| Right insula (13) | | 40 | | -21 | | 6 | 6.52 | | 6.33 | | 666 | |
| Left paracentral lobule (5) | | –8 | | –43 | | 55 | 6.20 | | 6.04 | | 250 | |
| Left insula | | -38 | | -21 | | 6 | 6.17 | | 6.02 | | 623 | |
| Right and left precuneus (31) | | 8 | | –66 | | 24 | 6.00 | | 5.85 | | 476 | |
|  |  | –4 | | –72 | | 25 | 5.27 | | 5.17 | |  |  |
| Right cerebellum | | 10 | | -39 | | -15 | 5.75 | | 5.63 | | 315 | |
| Right claustrum | | 38 | | 21 | | -3 | 5.18 | | 5.09 | | 65 | |
| Right parahippocampal gyrus (34) | | 16 | | –12 | | -17 | 5.12 | | 5.03 | | 47 | |
| Left inferior parietal lobule (40) | | –38 | | –48 | | 43 | 5.06 | | 4.97 | | 44 | |
| *Regions correlated with Free and Cued Selective Reminding Test (total recall)* | | | | | | | | | | | | |
| Left thalamus | | –20 | | –33 | | 1 | 5.90 | | 5.76 | | 577 | |
|  |  | 0 | | -21 | | 13 | 5.64 | | 5.52 | |  |  |
| Right thalamus | | 18 | | –31 | | 4 | 5.27 | | 5.17 | | 130 | |
| Right caudate | | 10 | | 14 | | 12 | 5.06 | | 4.97 | | 86 | |
| *Regions correlated with Free and Cued Selective Reminding Test (delayed free recall)* | | | | | | | | | | | | |
| Left and right thalamus, left and right caudate | | 0 | | -18 | | 13 | 8.16 | | 7.81 | | 14208 | |
|  |  | –16 | | –31 | | 4 | 7.71 | | 7.41 | |  |  |
|  |  | -15 | | -28 | | 12 | 7.68 | | 7.39 | |  |  |
| Right insula | | 39 | | –21 | | 6 | 5.65 | | 5.53 | | 203 | |
| Left parahippocampal gyrus (34) | | –14 | | –10 | | –15 | 5.33 | | 5.23 | | 44 | |
| Left insula | | -39 | | -19 | | 6 | 5.26 | | 5.16 | | 112 | |
| Right precuneus (31) | | 9 | | –64 | | 22 | 5.21 | | 5.11 | | 62 | |
| *Regions correlated with Free and Cued Selective Reminding Test (delayed total recall)* | | | | | | | | | | | | |
| Right thalamus | | 18 | | -31 | | 3 | 6.18 | | 6.02 | | 581 | |
| Left thalamus | | –18 | | –33 | | 1 | 5.98 | | 5.84 | | 877 | |
|  |  | –2 | | -21 | | 12 | 5.35 | | 5.25 | |  |  |
|  |  | -6 | | -34 | | 0 | 5.30 | | 5.20 | |  |  |
| Left caudate | | –4 | | 18 | | 4 | 5.30 | | 5.20 | | 237 | |
| *Regions correlated with verbal fluency (animals)* | | | | | | | | | | | | |
| Left and right caudate, left and right putamen | | 8 | | 12 | | 10 | 7.91 | | 7.60 | | 13774 | |
|  |  | –4 | | 12 | | 9 | 7.44 | | 7.18 | |  |  |
|  |  | 0 | | -9 | | 10 | 6.89 | | 6.67 | |  |  |
| Right and left thalamus | | 18 | | –33 | | 3 | 5.84 | | 5.70 | | 641 | |
| Left cerebellum (anterior lobe, culmen) | | –28 | | –49 | | –24 | 5.08 | | 4.99 | | 51 | |
| Right posterior cingulate (31) | | 9 | | -61 | | 16 | 4.98 | | 4.89 | | 36 | |
| *Regions correlated with verbal fluency (words beginning with “p”)* | | | | | | | | | | | | |
| Right caudate | | 20 | | 21 | | 3 | 5.49 | | 5.37 | | 609 | |
|  |  | 12 | | 18 | | 10 | 5.20 | | 5.11 | |  |  |
| *Regions correlated with verbal fluency (words beginning with “m”)* | | | | | | | | | | | | |
| Right caudate | | 18 | | 20 | | 1 | 6.32 | | 6.15 | | 2063 | |
|  |  | 10 | | 16 | | 9 | 6.12 | | 5.97 | |  |  |
| Left caudate | | –8 | | 15 | | 10 | 5.91 | | 5.77 | | 970 | |
| Left thalamus | | 0 | | -16 | | 15 | 5.09 | | 5.00 | | 59 | |
| *Regions correlated with verbal fluency (words beginning with “r”)* | | | | | | | | | | | | |
| Right caudate | | 16 | | 16 | | 4 | 5.69 | | 5.57 | | 1167 | |
| Left caudate | | –12 | | 16 | | 9 | 5.49 | | 5.38 | | 704 | |
| Left precuneus | | –6 | | –46 | | 48 | 5.16 | | 5.07 | | 95 | |
| *Regions correlated with Boston Naming Test* | | | | | | | | | | | | |
| Left and right thalamus | | 20 | | –28 | | 3 | 6.64 | | 6.44 | | 5512 | |
|  |  | 2 | | –22 | | 13 | 6.35 | | 6.18 | |  |  |
|  |  | 9 | | 16 | | 10 | 6.33 | | 6.16 | |  |  |
| Left caudate | | –3 | | 14 | | 4 | 5.99 | | 5.85 | | 813 | |
| Left parahippocampal gyrus (34) | | –15 | | 0 | | –15 | 5.52 | | 5.41 | | 112 | |
| Right parahippocampal gyrus (34) and hippocampus | | 26 | | –10 | | –18 | 5.62 | | 5.50 | | 524 | |
| Left hippocampus | | –27 | | –10 | | –20 | 5.26 | | 5.16 | | 54 | |
| *Regions correlated with Judgement of Line Orientation* | | | | | | | | | | | | |
| No suprathreshold clusters | | | | | | | | | | | | |
| *Regions correlated with Judgement of Line Orientation (&)* | | | | | | | | | | | | |
| Left thalamus | | –16 | | -30 | | 12 | 4.63 | | 4.56 | | 536 | |
| Left posterior cingulate and cuneus (18, 31) | | -9 | | -66 | | 10 | 4.30 | | 4.24 | | 1052 | |
|  |  | -3 | | -78 | | 19 | 3.46 | | 3.43 | |  |  |
| Left and right posterior cingulate (31) | | 9 | | -36 | | 39 | 4.21 | | 4.16 | | 639 | |
|  |  | -6 | | -34 | | 40 | 3.74 | | 3.70 | |  |  |
| Right inferior frontal gyrus and caudate (47) | | 27 | | 10 | | -17 | 4.10 | | 4.05 | | 2345 | |
|  |  | 27 | | 21 | | 0 | 4.04 | | 3.99 | |  |  |
|  |  | 12 | | 14 | | 12 | 3.71 | | 3.67 | |  |  |
| Left middle temporal gyrus (22) | | -48 | | -42 | | 9 | 4.09 | | 4.04 | | 286 | |
| Left angular and supramarginal gyri (39, 40) | | –50 | | –54 | | 28 | 4.03 | | 3.98 | | 267 | |
|  |  | –52 | | –63 | | 33 | 3.26 | | 3.24 | |  |  |
| Right thalamus | | 16 | | -28 | | 12 | 4.02 | | 3.97 | | 863 | |
| Left inferior frontal gyrus (47) and insula | | –40 | | 23 | | –9 | 4.02 | | 3.93 | | 413 | |
|  |  | -28 | | 32 | | -9 | 3.55 | | 3.52 | |  |  |
|  |  | -42 | | 29 | | -3 | 3.49 | | 3.45 | |  |  |
| Right cuneus and precuneus (7, 30, 31) | | 10 | | -60 | | 10 | 3.82 | | 3.78 | | 697 | |
|  |  | 22 | | -61 | | 30 | 3.50 | | 3.46 | |  |  |
|  |  | 8 | | -63 | | 21 | 3.40 | | 3.37 | |  |  |
| Left inferior parietal lobule (40) | | 40 | | –31 | | 36 | 3.69 | | 3.65 | | 199 | |
| Left caudate and putamen | | –14 | | 14 | | -3 | 3.67 | | 3.64 | | 2072 | |
|  |  | –6 | | 10 | | 0 | 3.59 | | 3.56 | |  |  |
|  |  | –22 | | 15 | | 3 | 3.57 | | 3.54 | |  |  |
| Left inferior frontal gyrus (9) | | –33 | | 9 | | 27 | 3.59 | | 3.56 | | 120 | |
| Left cerebellum (anterior lobe, culmen) | | –28 | | –49 | | –24 | 3.53 | | 3.50 | | 185 | |
| Right insula (13, 22) | | 38 | | -16 | | 13 | 3.49 | | 3.46 | | 200 | |
|  |  | 42 | | -22 | | 4 | 3.33 | | 3.30 | |  |  |
| Right middle temporal gyrus (21) | | 64 | | –49 | | 6 | 3.43 | | 3.490 | | 111 | |
| *Regions correlated with Rey-Osterrieth Complex Figure (copy accuracy)* | | | | | | | | | | | | |
| Right precentral gyrus (6) | | 63 | | –4 | | 16 | 5.22 | | 5.12 | | 80 | |
| *Regions correlated with Rey-Osterrieth Complex Figure (copy accuracy) (&)* | | | | | | | | | | | | |
| Right precentral gyrus (6) | | 63 | | –4 | | 16 | 5.22 | | 5.12 | | 1979 | |
| Left precentral and inferior frontal gyri (4, 9) | | –57 | | –4 | | 24 | 4.75 | | 4.68 | | 1763 | |
|  |  | –56  5 | | –9 | | 34 | 4.75 | | 4.67 | |  |  |
|  |  | –51 | | 6 | | 33 | 3.40 | | 3.337 | |  |  |
| Left inferior parietal lobule (40) | | –52 | | –60 | | 36 | 4.36 | | 4.30 | | 2679 | |
|  |  | -48 | | -43 | | 48 | 4.30 | | 4.24 | |  |  |
|  |  | –56 | | –33 | | 45 | 4.10 | | 4.05 | |  |  |
| Right precentral gyrus (4,6) | | 22 | | –13 | | 67 | 4.27 | | 4.21 | | 745 | |
|  |  | 40 | | -10 | | 54 | 3.82 | | 3.78 | |  |  |
|  |  | 36 | | -19 | | 63 | 3.57 | | 3.53 | |  |  |
| Left precentral gyrus (44) | | –51 | | 14 | | 4 | 3.91 | | 3.87 | | 232 | |
| Left superior and middle temporal gyri (21, 22) | | –69 | | –31 | | –6 | 3.88 | | 3.84 | | 382 | |
|  |  | –57 | | –25 | | -2 | 3.25 | | 3.23 | |  |  |
| Left middle temporal gyrus (21) | | –51 | | 9 | | –29 | 3.77 | | 3.73 | | 137 | |
| Left superior frontal gyrus (6) | | –4 | | 3 | | 66 | 3.68 | | 3.64 | | 144 | |
| Right medial frontal and cingulate gyrus (6, 24) | | 2 | | 3 | | 52 | 3.67 | | 3.63 | | 409 | |
|  |  | 2 | | -1 | | 42 | 3.48 | | 3.45 | |  |  |
|  |  | -3 | | -6 | | 57 | 3.33 | | 3.30 | |  |  |
| Left precentral gyrus (4) | | –30 | | –27 | | 63 | 3.53 | | 3.50 | | 101 | |
| Right postcentral gyrus (3) | | 38 | | –33 | | 62 | 3.49 | | 3.46 | | 207 | |
| Right superior and medial frontal gyri (9) | | 3 | | 47 | | 30 | 3.42 | | 3.39 | | 138 | |
|  |  | 3 | | 54 | | 24 | 3.37 | | 3.34 | |  |  |
| *Regions correlated with Rey-Osterrieth Complex Figure (recall at 3 minutes)* | | | | | | | | | | | | |
| Left thalamus | | -3 | | -4 | | 3 | 6.12 | | 5.96 | | 2109 | |
|  |  | –15 | | –33 | | 9 | 6.02 | | 5.87 | |  |  |
|  |  | –2 | | –21 | | 13 | 5.57 | | 5.44 | |  |  |
| Right thalamus | | 15 | | -33 | | 7 | 5.60 | | 5.47 | | 549 | |
| *Regions correlated with Rey-Osterrieth Complex Figure (recall at 30 minutes)* | | | | | | | | | | | | |
| Left thalamus | | –16 | | –31 | | 7 | 6.19 | | 6.02 | | 1575 | |
|  |  | –3 | | -4 | | 3 | 6.03 | | 5.88 | |  |  |
|  |  | –2 | | –18 | | 12 | 5.35 | | 5.24 | |  |  |
| Right thalamus | | 16 | | –31 | | 6 | 5.85 | | 5.71 | | 539 | |
| Left caudate | | –14 | | 20 | | -5 | 5.06 | | 4.96 | | 69 | |
| *Regions correlated with Rey-Osterrieth Complex Figure (recognition)* | | | | | | | | | | | | |
| Left caudate | | –6 | | 16 | | 7 | 5.31 | | 5.21 | | 316 | |
|  |  | –12 | | 21 | | 4 | 5.22 | | 5.12 | |  |  |
| Right thalamus | | 16 | | –33 | | 3 | 5.23 | | 5.12 | | 172 | |
| Left thalamus | | -14 | | -28 | | 13 | 5.14 | | 5.05 | | 81 | |
| Right inferior parietal lobe (40) | | 34 | | –34 | | 48 | 5.12 | | 5.02 | | 48 | |
| Left para-hippocampal gyrus (30) | | –15 | | -34 | | -3 | 5.09 | | 5.00 | | 58 | |
