# Supplementary material for: Identification of Cortical and Subcortical Correlates of Cognitive Performance in Multiple Sclerosis Using Voxel-Based Morphometry
Source: Front Neurol. 2018 Oct 29;9:920. doi: 10.3389/fneur.2018.00920 (PMC6216547; doi:10.3389/fneur.2018.00920)
Supplement: Supplementary file 4 [file Table_4.docx]

| **Table S4.** Correlation between brain regions and neuropsychological tests  Right hemisphere: blue; left hemisphere: yellow; bilaterally: green.  T: thalamus; C: caudate; Pt: putamen; AC: anterior cingulate and medial frontal gyrus; IF: inferior frontal gyrus; MF: middle frontal gyrus; SF: superior frontal gyrus; I: insula; ST: superior temporal; MT: middle temporal gyrus; Ph: parahippocampal gyrus; H: hippocampus; Prc: precentral gyrus; Pc: posterior cingulate and precuneus; Cn: cuneus; Sm: supramarginal and angular gyri; IP: inferior parietal lobule; MO: middle occipital gyrus; Cb: cerebellum.  (*): Using an uncorrected p-value <0.001 as threshold. | | | | | | | | | | | | | | | | | | |  |
| --- | --- | --- | --- | --- | --- | --- | --- | --- | --- | --- | --- | --- | --- | --- | --- | --- | --- | --- | --- |
|  | **T** | **C** | **Pt** | **AC** | **IF** | **MF** | **SF** | **I** | **ST** | **MT** | **Ph** | **H** | **Prc** | **Pc** | **Cn** | **Sm** | **IP** | **MO** | **Cb** |
| ***Corsi forward*** |  |  |  |  |  |  |  |  |  |  |  |  |  |  |  |  |  |  |  |
| ***Corsi backward*** |  |  |  |  |  |  |  |  |  |  |  |  |  |  |  |  |  |  |  |
| ***TMT-A*** |  |  |  |  |  |  |  |  |  |  |  |  |  |  |  |  |  |  |  |
| ***TMT-B*** |  |  |  |  |  |  |  |  |  |  |  |  |  |  |  |  |  |  |  |
| ***SDMT*** |  |  |  |  |  |  |  |  |  |  |  |  |  |  |  |  |  |  |  |
| ***Stroop A*** |  |  |  |  |  |  |  |  |  |  |  |  |  |  |  |  |  |  |  |
| ***Stroop B*** |  |  |  |  |  |  |  |  |  |  |  |  |  |  |  |  |  |  |  |
| ***Stroop C*** |  |  |  |  |  |  |  |  |  |  |  |  |  |  |  |  |  |  |  |
| ***ToL**** |  |  |  |  |  |  |  |  |  |  |  |  |  |  |  |  |  |  |  |
| ***FCSRT-FR1*** |  |  |  |  |  |  |  |  |  |  |  |  |  |  |  |  |  |  |  |
| ***FCSRT-TFR*** |  |  |  |  |  |  |  |  |  |  |  |  |  |  |  |  |  |  |  |
| ***FCSRT-TR*** |  |  |  |  |  |  |  |  |  |  |  |  |  |  |  |  |  |  |  |
| ***FCSRT-DFR*** |  |  |  |  |  |  |  |  |  |  |  |  |  |  |  |  |  |  |  |
| ***FCSRT-DTR*** |  |  |  |  |  |  |  |  |  |  |  |  |  |  |  |  |  |  |  |
| ***VF-animals*** |  |  |  |  |  |  |  |  |  |  |  |  |  |  |  |  |  |  |  |
| ***VF–p words*** |  |  |  |  |  |  |  |  |  |  |  |  |  |  |  |  |  |  |  |
| ***VF–m words*** |  |  |  |  |  |  |  |  |  |  |  |  |  |  |  |  |  |  |  |
| ***VF–r words*** |  |  |  |  |  |  |  |  |  |  |  |  |  |  |  |  |  |  |  |
| ***BNT*** |  |  |  |  |  |  |  |  |  |  |  |  |  |  |  |  |  |  |  |
| ***JLO**** |  |  |  |  |  |  |  |  |  |  |  |  |  |  |  |  |  |  |  |
| ***ROCF copy**** |  |  |  |  |  |  |  |  |  |  |  |  |  |  |  |  |  |  |  |
| ***ROCF–3 min*** |  |  |  |  |  |  |  |  |  |  |  |  |  |  |  |  |  |  |  |
| ***ROCF–30 min*** |  |  |  |  |  |  |  |  |  |  |  |  |  |  |  |  |  |  |  |
| ***ROCF-recog.*** |  |  |  |  |  |  |  |  |  |  |  |  |  |  |  |  |  |  |  |
| TMT: Trail Making Test; SDMT: Symbol Digit Modalities Test; ToL: Tower of London; FCSRT: Free and Cued Selective Reminding Test; VF: Verbal fluency; BNT: Boston Naming Test; JLO: Judgement of Line Orientation; ROCF: Rey-Osterrieth Complex Figure. | | | | | | | | | | | | | | | | | | | |
